# Effects of the activated mitogen-activated protein kinase pathway via the *c-ros* receptor tyrosine kinase on the T47D breast cancer cell line following alcohol exposure

**DOI:** 10.3892/or.2012.2209

**Published:** 2012-12-24

**Authors:** HYUNG TAE LEE, SE KYE KIM, MI RAN CHOI, JI HYUN PARK, KYOUNG HWA JUNG, YOUNG GYU CHAI

**Affiliations:** Department of Molecular and Life Science, Hanyang University, Ansan, Republic of Korea

**Keywords:** breast cancer, alcohol, receptor tyrosine kinase, mitogen-activated protein kinase pathway, histone phosphorylation

## Abstract

Compared to other cancers affecting women, breast cancer is significantly associated with alcohol consumption. However, the principles underlying the carcinogenesis of alcohol-induced breast cancer and the related metastatic mechanisms have yet to be established. To observe the effect of alcohol on the growth regulation in breast cancer cells, we identified differentially expressed proteins in alcohol-exposed human breast cancer T47D cells using gel-based proteomics analysis. The expression of *c-ros* receptor tyrosine kinase (ROS1) was increased and activated by autophosphorylation, thereby activating mitogen- and stress-activated protein kinase 1 (MSK1) through the mitogen-activated protein kinase (MAPK) pathway; activated MSK1, in turn, phosphorylated histone 3 serine 10 (H3S10p) residues in the nucleus. The increase in H3S10 phosphorylation consequently increased the level of expression of immediate-early gene such as *c-fos*. This study demonstrated that when breast cancer cells are exposed to alcohol, phosphorylated ROS1 activates MSK1 via ERK1/2 in the MAPK pathway, which then induces modifications to histone residues that regulate gene expression by 14-3-3 protein recruitment, leading to a lack of control of breast cancer cell proliferation.

## Introduction

Alcohol consumption is linked to cancer development and is considered to be the cause of certain types of cancers, including oropharynx, larynx, esophageal, liver, breast, colon and rectal cancers (1,2). Although many factors contribute to cancer in women, approximately 60% of alcohol-induced cancers in women are breast cancer (3). Various mechanisms, such as mutagenic effects, changes in retinoic acid, folate or estrogen metabolism and oxidative stress induced by alcohol consumption have been reported to be involved in breast cancer. However, the effects of alcohol on the carcinogenesis and metastasis of breast cancer have yet to be determined (2).

Alcohol affects both the upstream and downstream elements within the mitogen-activated protein kinase (MAPK) pathway in many tissue types (4). MAPK plays a role in relaying cellular responses to the nucleus; such responses include proliferation, differentiation, survival and apoptosis. MAPK can activate parallel cascades with such downstream targets as extracellular signal-regulated kinases 1/2 (ERK1/2), p38 MAPK and stress-activated protein kinase/c-Jun NH2-terminal kinase (SAPK/JNK). ERK1/2 is activated by receptor tyrosine kinases (RTKs) in response to alcoholic stress; it relays signals to the nucleus. In previous studies, the activity of ERK1/2 was found to be higher in breast cancer tissue in comparison to normal tissue (4–6). Increases in cell proliferation induced by activated ERK1/2 in response to alcohol have been observed in the human breast cancer cell line MCF-7 (7).

Epigenetic histone modification was recently reported as a carcinogenic mechanism induced by alcohol consumption (8,9). Histone modification affects gene expression by remodeling the chromatin structure. Histones are exposed to post-translational modifications, such as phosphorylation, acetylation, methylation, ubiquitination and SUMOylation, which regulate the activity of transcription factors, nucleosome remodelers, histone chaperones and other histone modifiers (10–12). The phosphorylation of histones regulates cellular processes, including transcription, DNA repair, chromosome condensation and apoptosis. For instance, the phosphorylation of histone 3 serine 10 (H3S10p) regulates the transcriptional activities of various genes during the interphase stage of the cell cycle, and the crosstalk with other modifications on the H3 tail residues (methylation, phosphorylation and acetylation) aids in the initiation of transcription (13). The activation of the MAPK pathway by UV irradiation increases the level of H3S10p and H3 serine 28 phosphorylation (H3S28p) (14,15). Rat hepatocytes exposed to alcohol and acetaldehyde also showed increases in the levels of H3S10p and H3S28p (16). Acute alcohol exposure also resulted in the same pattern of H3S10p and H3S28p levels, which, in turn, regulates the expression of *c-fos*, *c-jun* and *MKP-1*(9).

The following are known kinases that phosphorylate histones: Aurora B, VRK1, mitogen- and stress-activated kinase-1/2 (MSK1/2), PIM1, Rsk2, PKB/Akt and IKKα (13). MSK1/2, an H3 kinase, is activated by stress-activated ERK1/2 (17,18), and the resulting phosphorylation of H3 leads to the regulation of the expression of immediate-early (IE) genes, such as *c-fos* and *c-jun*(19–22). Activated by extracellular signals, the MAPK pathway was found to regulate alcohol-induced stress in cells, influencing the chromatin structure and gene expression (4). The present study analyzed the effect of alcohol on breast cancer cell proliferation through proteome profiling and observed the changes in histone modification patterns induced by signals relayed through the MAPK pathway in alcohol-treated breast cancer cells.

## Materials and methods

### Cell culture

Human ductal breast carcinoma T47D (KCLB No. 30133) cells were obtained from the Korean Cell Line Bank (Seoul, Korea). The T47D cells were grown in RPMI-1640 medium (Invitrogen, Carlsbad, CA, USA) supplemented with 10% fetal bovine serum (Invitrogen) and penicillin (100 U/ml)/streptomycin (100 μg/ml) (Invitrogen) at 37°C in a 5% CO_2_ atmosphere. The T47D cells were exposed for 48 h to 100 mM alcohol and 10 μM U0126 (MEK1/2 inhibitor), and the medium with alcohol was changed every 24 h. To prevent the evaporation of the alcohol from the culture dishes, the alcohol-treated cells were cultured in a separate CO_2_ incubator at the same alcohol concentration, as previously described (23).

### Proteomic analysis

To analyze the protein expression profile in T47D cells in the absence or presence of alcohol, we used the following proteomic techniques: two-dimensional gel electrophoresis (2-DE), matrix-assisted laser-desorption ionization time-of-flight mass spectrometry (MALDI-TOF MS) and database searches, as previously described by Jung *et al*(24). The protein samples for the 2-DE were extracted in lysis buffer [9.5 M urea, 2.5% 3-[(3-cholamidopropyl) dimethylammonio]-1-propanesulfonate (CHAPS), 40 mM dithiothreitol (DTT), 0.12% carrier ampholytes and 0.0012% bromophenol blue] and were normalized using the Bradford assay. Each sample (100 μg) was analyzed using immobilized pH gradient (IPG) DryStrips (pH 4.0–7.0) (Bio-Rad Laboratories, Hercules, CA, USA) for isoelectric focusing (IEF) and SDS-polyacrylamide gels. The IEF was performed at 20°C using a Protean^®^ IEF Cell (Bio-Rad Laboratories) following the manufacturer’s instructions. After electrophoresis, the 2-DE gels were stained with Coomassie brilliant blue and were analyzed to quantify the spot densities using PDQuest software (version 7.3, Bio-Rad Laboratories). Following the quantitative analysis, the differentially expressed protein spots were extracted from each gel. In-gel digestion was performed on selected protein spots, and the peptides were analyzed using an Ultraflex MALDI-TOF MS (Bruker Daltonics, Bremen, Germany) using a procedure similar to that previously described (24,25). The search program ProFound developed by Rockefeller University (http://prowl.rockefeller.edu/prowl-cgi/profound.exe) was used for the protein identification.

### Western blotting

The extraction of proteins from the cells was performed using RIPA buffer [1% Triton X-100 in 50 mM phosphate buffer (pH 7.4)] containing both a complete EDTA-free protease inhibitor cocktail (Roche Diagnostics, Mannheim, Germany) and a phosphatase inhibitor (GenDEPOT, Barker, TX, USA). The extracted proteins were separated on SDS polyacrylamide gels and transferred to polyvinylidene difluoride (PVDF) membranes (Schleicher & Schuell BioScience, Inc., Keene, NH, USA). The western blot analysis was performed using an anti-ROS1 antibody (Cell Signaling, Danvers, MA, USA), anti-phospho-ROS1 (Tyr2274) antibody (Cell Signaling), the phospho-ERK1/2 Pathway Sampler kit (Cell Signaling), anti-β-actin antibody (Sigma-Aldrich, St. Louis, MO, USA), anti-histone H3 (phospho-S10) antibody (Abcam, Cambridge, UK) and anti-histone H3 antibody (Abcam).

### Real-time reverse transcriptase (RT)-PCR

Total RNA was isolated from the T47D cells exposed or unexposed to alcohol and/or U0126 using TRIzol (Invitrogen). The total RNA was reverse-transcribed into cDNA using PrimeScript™ Reverse Transcriptase (Takara, Shinga, Japan), and the real-time PCR was performed using the 7500 Real-Time PCR system (Applied Biosystems, Foster City, CA, USA) and 2X SYBR-Green PCR Master mix (Takara). The sequences of the primers used in this study were as follows: *c-fos* forward, 5′-GTCTCCAGTG CCAACTTCATT-3′, and reverse, 5′-CCTCCTGTCATGG TCTTCACA-3′; and *β-actin* forward, 5′-TGGAGAAAATCT GGCACCACACC-3′, and reverse, 5′-GATGGGCACAGT GTGGGTGACCC-3′. *β-actin* was used as an internal control. The gene expression levels were analyzed using the 2^-ΔΔCT^ method (26).

### Determination of cell proliferation

The proliferation of the cells was evaluated using WST-1 (Takara) after exposure to 100 mM alcohol, 10 μM U0126 (Cell Signaling) for 12 and 24 h. The WST-1 reagent was added to each well, and the cells were incubated at 37°C in a 5% CO_2_ atmosphere for 4 h. The results of the WST-1 assay were measured using a Model 680 microplate reader (Bio-Rad Laboratories) at 440 nm.

### Chromatin immunoprecipitation (ChIP) assay

ChIP assay was performed as previously described by Choe *et al* with minor modifications (27). T47D cells were treated with 1% formaldehyde for 10 min at 37°C. After harvesting, 2×10^7^ cells were suspended with Tris-EDTA buffer (10 mM Tris-HCl, pH 7.6, 1 mM EDTA) including 5 mM butyrate, 1X proteinase inhibitor cocktail (Roche Diagnostics) and 0.5 mM fresh PMSF. After sonication, the cells were dialyzed with RIPA buffer (10 mM Tris, pH 7.4, 1 mM EDTA, 0.1% SDS, 0.1% sodium deoxycholate, 1% Triton X-100) and subject to immunoprecipitation with antibodies against anti-14-3-3ɛ, anti-14-3-3ζ and IgG (Santa Cruz Biotechnology, Santa Cruz, CA, USA). Isolated chipped DNA was validated by PCR. The sequences of primers for the 14-3-3 protein recruitment assay are listed in Table I.

### Statistical analysis

All of the values were analyzed using OriginPro 8 (OriginLab Corp., Northampton, MA, USA). All of the values are expressed as the mean ± standard error of the mean (SEM). All of the statistical analyses were performed using SPSS 17.0 (SPSS Inc., Chicago, IL, USA). P-values <0.05 were considered to indicate a statistically significant difference.

## Results

### Identification of upregulated ROS1 protein in alcohol-treated T47D cells by proteomic analysis

**Figure 1:**
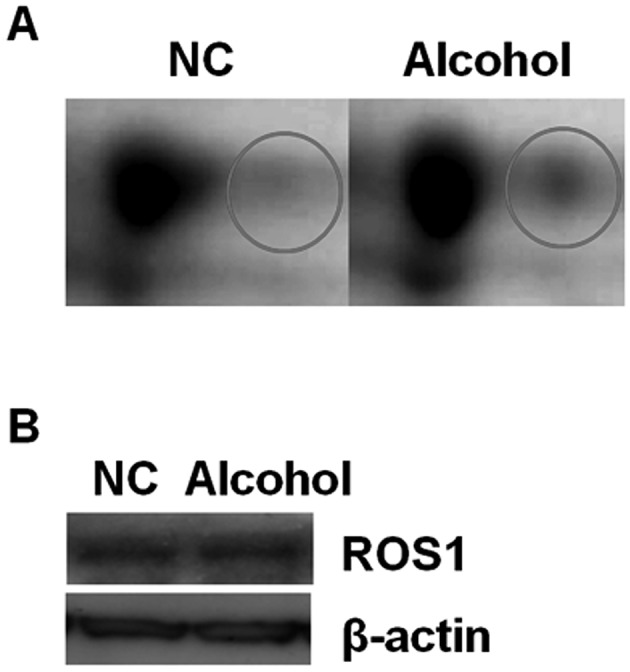
Expression pattern of the ROS1 protein in breast cancer cells after alcohol exposure. T47D breast cancer cells were incubated in the presence or absence of 100 mM alcohol for 48 h. Equal amounts of proteins were obtained from the cells and were analyzed using (A) 2-DE and (B) western blotting. The expression of ROS1 was increased in the alcohol-exposed breast cancer cells. β-actin was used as an internal control.

To assay the influence of alcohol on breast cancer, proteins extracted from untreated and alcohol-exposed T47D cells (100 mM) for 48 h were isolated with 2-DE and identified using MALDI-TOF MS. A total of 20 proteins displaying more than a 2-fold difference in expression level were identified: 7 proteins increased in response to alcohol exposure, whereas 13 proteins displayed reductions in their expression levels (Tables II and III). ROS1, a RTK differentially induced by alcohol, demonstrated an increased expression ~2.3 times more than that under normal conditions in both 2-DE imaging (Fig. 1A) and western blotting (Fig. 1B).

### Effect of alcohol on the phosphorylation of ROS1 and the MAPK pathway

**Figure 2:**
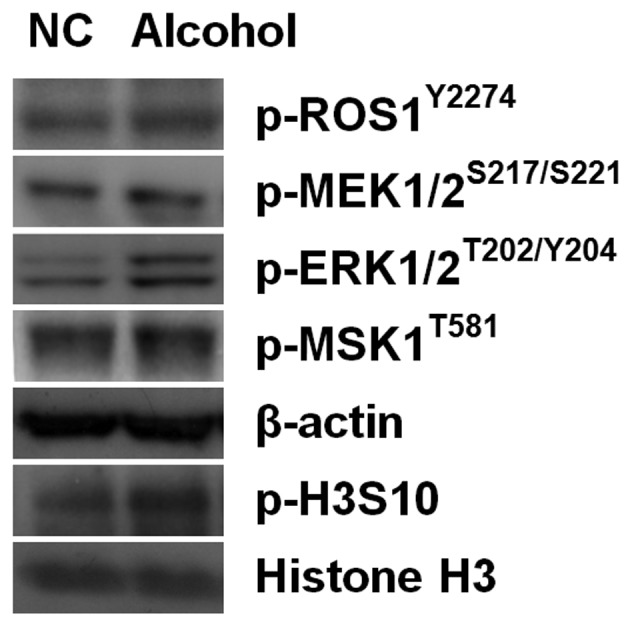
Regulation of the MAPK pathway and histone modification in response to alcohol. T47D cells were incubated in the presence or absence of 100 mM alcohol for 24 h. Equal amounts of proteins were obtained from these cells and were analyzed using western blotting to detect phosphorylated (p)-ROS1 (Y2274), p-ERK1/2 (T202/Y204), p-MSK1 (T581) and p-H3S10. The phosphorylation levels of ROS1, ERK1/2, MSK1 and H3S10 were significantly increased in the alcohol-exposed breast cancer cells. β-actin and histone H3 were used as internal controls. NC, untreated control.

Western blotting was performed to determine the activation of the ROS1 protein and MAPK pathway proteins by alcohol-induced phosphorylation (Fig. 2). The phosphorylation of the Y2274 residue on the ROS1 protein increased at 24 h after the exposure of T47D cells to 100 mM alcohol; the phosphorylation of MEK1/2, ERK1/2, MSK1 and H3S10 also showed similar patterns after alcohol treatment. These results suggest that the level of H3S10p was increased via activated MAPK pathways in alcohol-exposed T47D cells.

**Figure 3:**
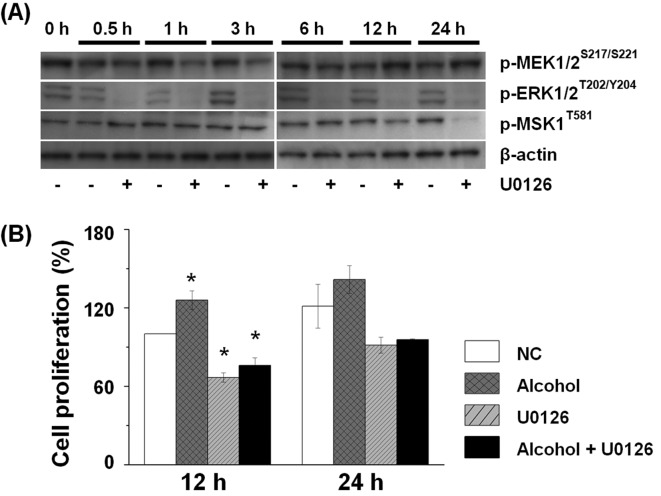
Exposure to alcohol resulted in the activation of the MAPK pathway and increased cell proliferation. (A) T47D cells were incubated in the presence or absence of 10 μM U0126, a MEK1/2 inhibitor, for 0.5, 1, 3, 6, 12 and 24 h. Equal amounts of proteins were obtained from the cells and were analyzed using western blotting to detect p-MEK1/2 (S217/S2221), p-ERK1/2 (T202/Y204) and p-MSK1 (T581). Phosphorylated MEK was decreased at 0.5, 1 and 3 h in the T47D cells exposed to alcohol in the presence of U0126. Phosphorylated ERK1/2 was decreased at 0.5, 1, 3, 6, 12 and 24 h in the T47D cells treated with alcohol in the presence of U0126. Phosphorylated MSK was decreased at 12 and 24 h in the T47D cells treated with alcohol in the presence of U0126. (B) The T47D cells were exposed to 100 mM alcohol and/or 10 μM U0126 for 12 and 24 h; the cell proliferation was evaluated, and the proliferation of the cells was measured using WST-1. The T47D cells treated with alcohol demonstrated an increase in cell proliferation and the U0126-treated cells showed a decrease compared with the untreated cells. However, exposure to both alcohol and U0126 induced an increase in cell proliferation when compared to the U0126-treated cells. The values are presented as means ± SEM (n=3). ^*^Significantly different from the NC group. A one-way ANOVA followed by Tukey’s HSD post-hoc test was used to evaluate the statistical significance of the differences. NC, untreated control.

To further investigate the role of alcohol in the activation of the MAPK pathway, cell proliferation was examined in the alcohol- and/or MEK1/2 inhibitor U0126-treated T47D cells. First of all, we assessed the optimal inhibition time of MAPK pathway by U0126 treatment in T47D cells. A gradual decrease in the level of the MEK1/2 phosphorylation was observed in the U0126-treated T47D cells at 1, 3 and 6 h, while it increased after 12 h. The phosphorylation of ERK1/2, on the other hand, was inhibited during the entire observation period and was not affected by activated MEK1/2. The phosphorylation level of MSK1, a kinase activated downstream of the MAPK cascade, was also reduced at 12 and 24 h (Fig. 3A).

Cell proliferation was evaluated after exposure to alcohol and/or U0126 at 12 and 24 h (Fig. 3B). When T47D cells were exposed to alcohol, the cell numbers exhibited a greater increase than that of the unexposed cells after 12 and 24 h of incubation. In contrast, when U0126 was added for 12 h, the number of cells was reduced to ~66%, and a slight increase in the number of cells was observed after 24 h. When T47D cells were exposed to both alcohol and U0126 for 12 and 24 h, the number of cells increased to a greater extent than that of the U0126-treated cells. It can be inferred that alcohol leads to an increase in cell proliferation through activation of the MAPK pathway.

### Regulation of immediate-early gene expression following exposure to alcohol and the MEK1/2 inhibitor treatment

**Figure 4:**
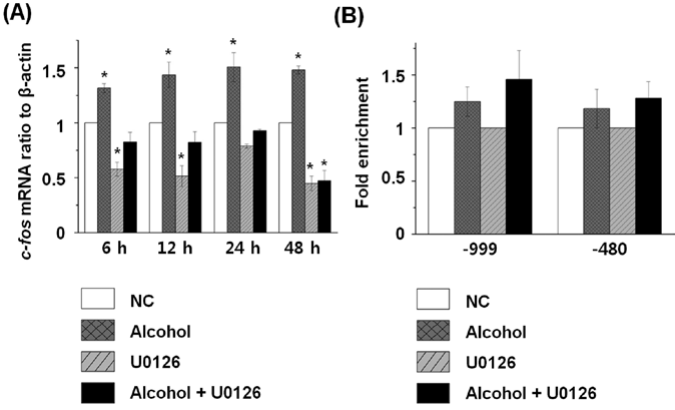
Alteration of immediate-early (IE) gene expression and 14-3-3 protein recruitment in response to alcohol. (A) The expression pattern of IE genes was analyzed using real-time RT-PCR. The mRNA level of *c-fos* was increased in the T47D cells exposed to alcohol. However, the mRNA level of *c-fos* was decreased in the T47D cells treated with U0126 in comparison to the T47D cells treated without alcohol (NC), but was increased in the T47D cells exposed to both alcohol and U0126 compared with the U0126-treated cells. (B) Chromatin immunoprecipitation (ChIP) experiments were performed using antibodies against 14-3-3ɛ and 14-3-3ζ. The T47D cells exposed to alcohol for 24 h were prepared by formaldehyde-crosslink. Enrichment values of 14-3-3 proteins obtained by PCR assays on equal amounts of immunoprecipitated DNA. The enrichment levels of 14-3-3 proteins were increased in the T47D cells exposed to alcohol in comparison to the T47D cells not exposed to alcohol (NC) at upstream regions (-999, -480) of the *c-fos* gene. *β-actin* was used as an internal control.

As cell proliferation was increased due to the activation of the alcohol-induced MAPK cascade, we analyzed the effect of alcohol and U0126 on relevant IE gene expression patterns (Fig. 4A). The expression of *c-fos*, one of the IE genes, was controlled by increases in H3S10p, as confirmed using real-time RT-PCR. The expression of *c-fos* was increased after exposure to alcohol during the entire exposure, while the expression level was reduced to half when U0126 was added. However, the expression level was slightly increased when both alcohol and U0126 were administered to T47D cells. These results indicate that the expression level of *c-fos* is increased according to elevated H3S10p through activation of the MAPK pathway in alcohol-exposed cells.

### Regulation of recruitment of the 14-3-3 proteins in response to alcohol exposure

In previous research, 14-3-3 proteins were reported to act as adaptors between phosphorylated histone H3 and another phosphoprotein (28). Additionally, recruitment of 14-3-3 proteins such as 14-3-3ɛ and 14-3-3ζ were found to be increased by ERK1/2 MAPK pathway activation for inducible genes (29). To determine the composition of 14-3-3 proteins of the *c-fos* gene after alcohol exposure in T47D cells, we performed a ChIP assay (Fig. 4B). Upon alcohol exposure, recruitment of 14-3-3 proteins was increased in both upstream regions (−999, −480) of the *c-fos* gene, indicating that recruitment of 14-3-3 proteins is induced after alcohol exposure at the upstream regions of *c-fos*.

## Discussion

In this study, we observed that the expression of ROS1 and its phosphorylation level were enhanced in alcohol-exposed T47D cells. ROS1, a proto-oncogene expressed in various tumor cell lines, is a cellular signaling transduction pathway regulator that mediates cell proliferation, migration and cell-to-cell communication (30,31). ROS1 is activated via the autophosphorylation of Y2274 and Y2334 residues and regulates signaling transduction pathways, e.g., the MAPK, insulin receptor substrate 1 (IRS-1), phosphatidylinositol 3-kinase (PI3K), protein kinase B (AKT), STAT3 and VAV3 signaling pathways (30).

Among these pathways, the MAPK pathway influences the chromatin structure and gene expression, thereby regulating cellular processes such as cell proliferation, apoptosis, inflammation and cell cycle progression (5,32,33). Cell proliferation is enhanced through the MAPK pathway in various tissues due to the chronic intake of alcohol (34–36). When 10–40 mM alcohol was added to human hepatocellular carcinoma cell lines HepG2 and SKHep, ERK1/2 was activated, and cell proliferation was enhanced, while the alcohol-induced proliferation was inhibited when a MEK1/2 inhibitor was added (37). ERK1/2, p38 MAPK and JNK were found to be activated in alcohol-exposed mouse hippocampal HT22 cells, and p38 MAPK activation was proposed to be related to the production of reactive oxygen species (38).

In many case studies, the activation of ERK1/2 in breast cancer cells is usually higher than that in normal cells. This observation is consistent with the results from comparative studies of primary breast tumors and nearby normal tissues in breast cancer patients in which the severity of symptoms is dependent on the level of ERK1/2 activation (4–6). ERK1/2 was activated by a pathophysiologically relevant concentration (65 mM) of alcohol in the human breast cancer cell line MCF-7, and activated ERK1/2-induced increases in cell proliferation up to 400%, but when an MEK1 inhibitor (PD98059) was administered with alcohol, the cell proliferation was reduced to 200% (7). Similar to MCF-7 and various tissue cells such as hepatocytes and hippocampal cells, the breast cancer cell line T47D exhibited an activation of ERK1/2 and MSK1 via phosphorylation when 100 mM alcohol was added. Furthermore, we demonstrated that the proliferation of T47D cells showed a greater increase when 100 mM alcohol was added when compared with untreated cells. In addition, cell proliferation was reduced in the MEK1/2 inhibitor U0126-treated T47D cells. However, we found that exposure to both alcohol and U0126 restored cell proliferation when compared with U0126-treated T47D cells. For these reasons, we suggest that alcohol-induced ROS1 activity influences MAPK pathway activation and cell proliferation.

Our results showed that the MSK1 phosphorylation level increased via the MAPK pathway in alcohol-exposed breast cancer cells and that phosphorylated MSK1 acts as an H3 kinase, increasing the level of H3S10p. A previous study showed that the level of H3S10p and H3S28p increased at 1–4 h after the intraperitoneal injection of 5 g/kg alcohol in rats, leading to the regulation of *c-fos*, *c-jun* and *MKP-1* expression (9). When 100 mM alcohol and 5 mM acetaldehyde were administered separately to rat hepatocytes, the phosphorylation level of p38 MAPK reached its peak at 24 h and 30 min, respectively, and the induced level of p38 MAPK increased the levels of H3S10p and H3S28p (16). Similar to previous results, we showed that histone phosphorylation is increased by alcohol exposure in breast cancer cells. Therefore, we confirmed that alcohol is closely related to histone phosphorylation.

In this study, we established that alcohol-induced H3S10p regulates the recruitment of 14-3-3 proteins at the upstream regions of *c-fos*. The 14-3-3 proteins including the isoforms ɛ and ζ, an abundant family of phospho-specific binding proteins, were reported to bind to phosphorylated histone residues such as serine 10 and 28 (28,39). In addition, it functions as a link between phosphorylated histone residues and 14-3-3 binding proteins (29). A previous study reported that phosphorylated H3S10 by TPA-stimulated MSK1 is recruited to 14-3-3 proteins mediating the recruitment of SWI/SNF, FOS/JUN and RNA polymerase II at the target gene promoter region finally inducing the expression of target IE genes (29). After alcohol exposure, we also observed that binding of 14-3-3 proteins to the upstream regions of the *c-fos* gene was increased and induced gene expression. Notably, all the events such as H3S10 phosphorylation, the recruitment of 14-3-3 proteins and the expression of the IE genes were reduced by MSK1 knockdown (29). Thus, we suggest that activated MSK1 by external stimuli including alcohol plays an important role in histone remodeling and IE gene expression.

When T47D human breast cancer cells are exposed to alcohol, the expression of ROS1 is upregulated, and autophosphorylated ROS1, in turn, activates MSK1 via ERK1/2 in the MAPK pathway. The MSK1-induced increase in H3S10p induces the expression of *c-fos* through 14-3-3 protein recruitment, influencing cell growth under alcohol-exposed conditions. This is the first study to show that activation of the ROS1 protein by alcohol exposure in breast cancer cells is associated with the MAPK pathway. Furthermore, this study demonstrated the relationship between alcohol and cell proliferation associated with activation of the MAPK pathway by ROS1 protein. Based on our results and previous studies, it is believed that breast cancer cells exposed to alcohol show increased cancer cell growth by activating the MAPK pathway through ROS1 protein, therefore confirming that alcohol consumption is detrimental to breast cancer patients.

## Figures and Tables

**Table I:** List of primer sequences used to amplify chipped DNA.

	Primer sequence 5′ to 3′	
	
	Forward	Reverse
*c-fos* (-999)	CGTGGTTGAGCCCGTGACGTT	TGCGGTTGGAGTACGAGGCG
*c-fos* (-480)	GGGCGGGACGCTCCAGTAGAT	TCAGAGCAAGTCCCGAGCCC
*GAPDH*	GCAAGGAGAGCTCAAGGTCA	AGCGCGAAAGGAAAGAAAG

**Table II:** Upregulated proteins in T47D cells exposed to alcohol in comparison to the unexposed cells.

	Intensity			
			
Protein	NC	Alcohol	Est’d Z	Accession number
Unnamed protein product	2,126.3	6,055.9	2.4	AAA73055.1
Nestin, isoform CRA_b	7,729.2	13,638.2	0.3	EAW52924.1
26S protease regulatory subunit 6B isoform 1	5,411.6	6,413.1	2.4	NP_006494.1
ATPase, H^+^ transporting, lysosomal 50/57 kDa, V1 subunit H, isoform CRA_c	5,748.3	10,912.2	2.43	EAW86730.1
RNA binding motif protein 4, isoform CRA_b	2,086.9	4,221.9	2.43	EAW74555.1
RUN and FYVE domain-containing protein 4	3,851.3	6,874.7	0.6	NP_940885.2
ROS1	4,316.6	10,284.5	1.43	AAA60277.1

Est’d Z score is a chance value used in ProFound that corresponds to the percentile of the search in a random-match population. For instance, a Z score of 1.65 for a search indicates that the search is in the 95th percentile. NC, untreated control.

**Table III:** Downregulated proteins in the T47D cells treated with alcohol in comparison to the untreated cells.

	Intensity			
			
Protein	NC	Alcohol	Est'd Z	Accession number
SRB7 suppressor of RNA polymerase B homolog (yeast), isoform CRA_a	7,694.7	3,585.2	1.4	EAW96544.1
CD44 molecule (Indian blood group)	2,125.9	1,339.9	1.4	CAC10349.1
G α-q	8,732	3,843.1	1.3	AAB06875.1
β-tubulin	4,769.7	1,569	2.4	AAB59507.1
Keratin, type I cytoskeletal 19	21,769.7	12,874.6	2.4	P08727.3
Keratin, type I cytoskeletal 19	20,520.4	2,340.1	2.4	P08727.3
RIN2 protein	10,761.2	3,308.4	0.9	AAI28066.1
Keratin, type I cytoskeletal 19	10,293.7	2,111.6	2.4	NP_002267.2
β-tubulin	11,069	4,955.7	2.4	AAB59507.1
HMMR protein	6,474	5,410.5	1.4	AAH06984.1
α-amylase	13,589.9	9,157.8	2.4	NP_004029.2
3-hydroxyisobutyrate dehydrogenase	1,523.5	279.9	1.9	NP_689953.1
Keratin, type II cytoskeletal 79	17,336.5	12,267.3	2.4	Q5XKE5.1

Est'd Z score is a chance value used in ProFound that corresponds to the percentile of the search in a random-match population. For instance, a Z score of 1.65 for a search indicates that the search is in the 95th percentile. NC, untreated control.
